# An allantoin-inducible glyoxylate utilization pathway in Pseudomonas aeruginosa

**DOI:** 10.1099/mic.0.001635

**Published:** 2025-12-10

**Authors:** Susannah L. Parkhill, Olivia Little, Isabel Askenasy, Edoardo Labrini, Meng Wang, Paul D. Brear, Wei Cai, Tomas Deingruber, Tianyi Yang, David R. Spring, Martin Welch

**Affiliations:** 1 Department of Biochemistry, University of Cambridge, Hopkins Building, Tennis Court Road, Cambridge, CB2 1QW, UK; 2 Yusuf Hamied Department of Chemistry, University of Cambridge, Lensfield Road, Cambridge, CB2 1EW, UK

**Keywords:** allantoin, glyoxylate, metabolism, *Pseudomonas aeruginosa*, purine catabolism

## Abstract

Fluorescent pseudomonads catabolize purines via uric acid and allantoin, a pathway whose end-product is glyoxylate. In this work, we show that in *Pseudomonas aeruginosa* strain PAO1, the ORFs PA1498–PA1502 encode a pathway that converts the resulting glyoxylate into pyruvate. The expression of this cluster of ORFs was stimulated in the presence of allantoin, and mutants containing transposon insertions in the cluster were unable to grow on allantoin as a sole carbon source. The likely operonic structure of the cluster is elucidated. We also show that the purified proteins encoded by PA1502 and PA1500 have glyoxylate carboligase (Gcl) and tartronate semialdehyde (TSA) reductase (GlxR) activity, respectively, *in vitro*. Gcl condenses two molecules of glyoxylate to yield TSA, which is then reduced by GlxR to yield d-glycerate. GlxR displayed much greater specificity (*k*_cat_/K_M_) for Gcl-derived TSA than it did for the TSA tautomer, hydroxypyruvate. This is relevant because TSA can potentially spontaneously tautomerize to yield hydroxypyruvate at neutral pH. However, kinetic and [^1^H]-NMR evidence indicate that PA1501 (which encodes a putative hydroxypyruvate isomerase, Hyi) increases the rate of the Gcl-catalysed reaction, possibly by minimizing the impact of this unwanted tautomerization. Finally, we use X-ray crystallography to show that apo-GlxR is a configurationally flexible enzyme that can adopt two distinct tetrameric assemblies *in vitro*.

## Introduction

*Pseudomonas aeruginosa* is a Gram-negative opportunistic pathogen, associated with severe respiratory infections, ventilator-associated pneumonia, urinary tract infections and other skin and soft tissue infections [1]. It possesses a versatile metabolism, which even now continues to reveal hitherto novel metabolic cycles and pathways [2]. *P. aeruginosa* does not encode phosphofructokinase and so cannot use the ‘classical’ Embden–Meyerhof–Parnas (EMP) pathway for glycolysis. Instead, the organism uses the more primitive Entner–Doudoroff pathway (EDP) for glucose metabolism [3]. Pyruvate kinase catalyses the final step of glycolysis in both the EDP and EMP and is thus a key point of regulation in many species [4,6]. *P. aeruginosa* encodes two pyruvate kinase isozymes (*pykF* and *pykA*): PykA appears to be the dominant isozyme in most growth conditions, but both isozymes display similar regulatory properties, leaving the biological function of PykF unclear [7,8]. However, *pykF* (PA1498) expression is induced in the presence of the diureide, allantoin, and a pronounced growth defect is observed during growth of a *pykF* mutant with allantoin as a sole carbon source, indicating a potential role for *pykF* in allantoin metabolism [7].

**Fig. 1.**
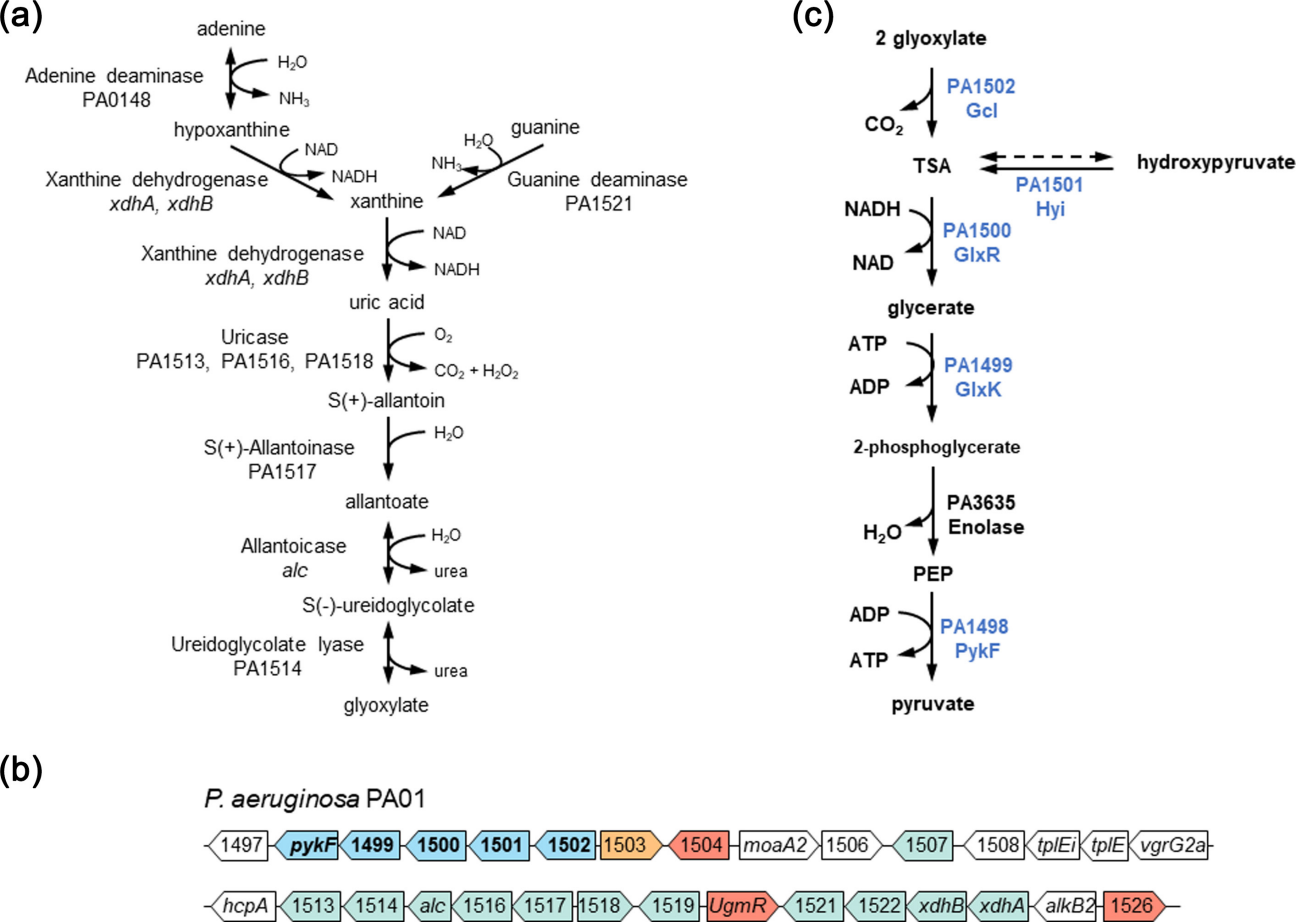
Allantoin metabolism in *P. aeruginosa*. (**a**) Putative pathway of purine degradation to glyoxylate in *P. aeruginosa*. Note that not all of the enzymes in this pathway have been biochemically characterized and are proposed based on amino acid similarity with those from other organisms. (**b**) Genetic context of the ORFs involved in purine degradation in *P. aeruginosa*. Blue, ORFs involved in the conversion of glyoxylate to 2-phosphoglycerate (investigated in the current work); green, ORFs predicted to encode enzymes involved in the purine/allantoin degradation pathway shown in (**a**); red, regulatory proteins; orange, ORFs encoding enzymes involved in glycolate oxidation; white, other. The two rows of ORFs represent one contiguous sequence. Diagram not to scale. (**c**) Proposed pathway of glyoxylate metabolism catalysed by the *gcl* cluster ORFs [shown in blue here and in (**b**)].

Allantoin is an intermediate in purine catabolism. Many (but not all) of the enzymatic activities required to convert purines to allantoin, and the subsequent degradation of allantoin to glyoxylate, have been demonstrated in extracts of *P. aeruginosa* [9,12]. This pathway (Fig. 1a) allows *P. aeruginosa* to utilize purines and their derivatives as a sole carbon source [9,13]. Before this can occur though, the pathway end product, glyoxylate, needs to be converted to an anabolically utilizable intermediate. Although the activities of enzymes potentially catalysing this aspect of glyoxylate metabolism have been demonstrated in cell extracts from *Pseudomonas putida* and some other unspecified pseudomonads, the enzymes themselves have not been characterized in *P. aeruginosa* [14,18]. Nevertheless, a gene cluster (PA1498-PA1502) in *P. aeruginosa* encoding candidate enzymes has been inferred on the basis of homology.

In addition to being derived from allantoin catabolism, glyoxylate is also produced during growth on fatty acid substrates by the action of *iso*citrate lyase on *iso*citrate. The glyoxylate thus produced then undergoes an aldol condensation with acetyl-CoA (catalysed by malate synthase G [19]) to yield a gluconeogenic precursor, malate. This so-called ‘glyoxylate shunt’ preserves carbon units for biomass production [20]. The malate synthase-catalysed step also provides a mechanism to prevent glyoxylate – a potentially toxic aldehyde – from accumulating in the cell. However, acetyl-CoA is not co-produced with glyoxylate during allantoin degradation, so the glyoxylate shunt avenue of detoxification is not appropriate. Instead, a specialist pathway is required. In *Escherichia coli*, this pathway involves three enzymes: glyoxylate carboligase, tartronate semialdehyde (TSA) reductase and glycerate kinase [21,22]. Collectively, these enzymes convert glyoxylate into the central metabolic intermediate, 2-phosphoglycerate. *P. aeruginosa* also encodes homologues of glyoxylate carboligase (PA1502), TSA reductase (PA1500) and glycerate kinase (PA1499). However, these three ORFs form part of a larger cluster, which also includes the previously characterized *pykF* (PA1498) [7] and a putative hydroxypyruvate isomerase (PA1501). We further note that this cluster of ORFs is located just upstream of PA1513–PA1518, which are predicted to encode enzymes in the ‘mainstream’ pathway of purine degradation (Fig. 1a, b). In the current study, we characterize the PA1498–PA1502 ORF cluster and examine the biochemical activity of key purified enzymes in the encoded pathway. Our data indicate that one of these ORFs (PA1501, encoding Hyi) likely evolved to minimize the impact of a potentially deleterious spontaneous side reaction. We also find that another of the enzymes, GlxR (PA1500), is configurationally flexible and can adopt more than one distinct tetrameric structure. Finally, we show that the expression of the cluster is strongly induced during growth on allantoin and that disruption of the ORFs by transposon insertions abolishes the ability to grow on allantoin as a sole carbon source. In summary, we show that in *P. aeruginosa*, the loci PA1498–PA1502 comprise a dedicated glyoxylate catabolic unit that converts allantoin-derived glyoxylate into the central metabolite, pyruvate (Fig. 1c).

## Methods

### Bacterial strains, media and growth conditions

The bacterial strains and plasmids used in this study are shown in Table S1 (available in the online Supplementary Material). *E. coli* and *P. aeruginosa* strains were routinely cultured in Luria-Bertani (LB) media (Lennox formulation) or in M9 minimal media supplemented with the indicated carbon source. Liquid cultures were grown with vigorous aeration (200 r.p.m.) at 37 °C. When required, antibiotics were used at the following concentrations: for *E. coli,* 10 µg ml^−1^ tetracycline, 50 µg ml^−1^ carbenicillin and 25 µg ml^−1^ chloramphenicol and for *P. aeruginosa*, 5 µg ml^−1^ (UWGC strains) or 50 µg ml^−1^ (transformant selection) tetracycline.

Transposon mutants containing either the IS*phoA*/hah or IS*lacZ*/hah transposon were obtained from the UWGC mutant bank and confirmed by PCR using the recommended primers [23]. For liquid cultures, overnight starters were pelleted and washed three times in sterile PBS and then used to inoculate 100 µl M9 minimal medium supplemented with 0.25% (w/v) glucose or 0.33% (w/v) allantoin to an initial OD_600_ of 0.05 in a round-bottomed 96-well plate. The plate was sealed with a gas-permeable membrane and incubated in an Omega FLUOstar plate reader, with OD_600_ measurements taken every 15 min for 20 h.

### Construction of P*_gcl_*-*lacZ* and P*_0_*-*lacZ* reporter strains

A 554 bp region upstream of *gcl* was PCR-amplified from PA01 genomic DNA (gDNA) using Phusion HiFi DNA polymerase (NEB) and oligonucleotide pair P_gcl_HindIIIF and P_gcl_EcoRIR (Table S2). The PCR product was digested with EcoRI and HindIII and ligated into similarly digested miniCTX*lacZ* plasmid [24] to produce miniCTX*lacZ*-P*_gcl_*. The miniCTX*lacZ*-P*_gcl_* was introduced into *E. coli* JM109 by electroporation and successful transformants were confirmed by colony PCR with oligonucleotide pair mcl3997F and mcl4255R (Table S2). The confirmed miniCTX*lacZ*-P*_gcl_* (designated P*_gcl_-lacZ*) and the empty miniCTX*lacZ* vector (a promoter-less *lacZ* control, designated P_0_*-lacZ*) were introduced into PA01 by electroporation. Successful integrants at the *attB* site were confirmed by colony PCR using oligonucleotide pairs (i) mcl3997F and mcl4255R, (ii) P_serup2_ and P_serdown2_, (iii) P_serup2_ and mcl961 and (iv) P_serdown2_ and mcl8810 (Table S2).

### *β*-Galactosidase activity

Samples of PAO1 containing P*_gcl_-lacZ* or P_0_*-lacZ* grown in M9-allantoin were taken at OD_600_ 0.2 (mid-log) and OD_600_ 0.4 (stationary phase), whereas those grown in M9-glucose were taken at OD_600_ 0.4 (mid-log) and OD_600_ 1 (stationary phase). The cells were pelleted (20,000 ***g***, 5 min, 4 °C) and then resuspended in 0.5 ml Z buffer (0.06 M Na_2_HPO_4_, 0.044 M NaH_2_PO_4_, 10 mM KCl, 1 mM MgSO_4,_ 0.27% (v/v) *β*-mercaptoethanol and pH 7). The OD_600_ of each sample was measured. Next, and to permeabilize the cells for *β*-galactosidase assay, 250 µl of cell resuspension was added to 750 µl Z buffer, 50 µl 0.1% (w/v) SDS and 100 µl chloroform. The mixture was vortexed and incubated (10 min, 30 °C) and then mixed with 100 µl of ONPG solution [4 mg ml^−1^ ONPG dissolved in 60 mM Na_2_HPO_4_ and 44 mM NaH_2_PO_4_ (pH 7.0)]. After 30 min (mid-log phase samples) or 2 h (stationary phase samples), the reaction was stopped by the addition of 400 µl 1 M Na_2_CO_3_. Cell debris was pelleted (20,000 ***g***, 10 min) and the supernatant (100 µl) was transferred to a flat-bottomed 96-well plate for A_420_ measurement (Omega FLUOstar). Three biological replicates were assayed for each strain at each time point. Statistical significance was analysed using unpaired t-tests using GraphPad Prism v10.

### Quantitative Reverse Transcription PCR (RT-PCR)

Total RNA was extracted from 4 ml samples of PAO1 collected during the exponential phase of growth (OD₆₀₀ = 0.2–0.4). Cultures were grown in 50 ml of M9 medium supplemented with allantoin at a concentration of 0.33% (w/v) in 250 ml flasks at 37 °C with vigorous aeration (120 r.p.m.). Cells were harvested by centrifugation, resuspended in Max Bacterial Enhancement Reagent and boiled at 95 °C for 10 min. TRIzol (Qiagen) and cold chloroform were then added to lyse the cells and induce phase separation. The samples were then centrifuged, and the RNA was partitioned into the upper aqueous phase. RNA was purified using the Monarch Spin RNA Cleanup Kit (NEB) according to the manufacturer’s instructions. cDNA was synthesized from 1 µg of total purified RNA using SuperScript III Reverse Transcriptase (Invitrogen) according to the manufacturer’s instructions. RT-PCR was performed using *Taq* DNA polymerase with Standard *Taq* Buffer (NEB), 3 µg of cDNA and the primers listed in Table S2. gDNA was used as a positive control at a concentration of 500 ng. PCR amplicons were resolved by electrophoresis on a 0.8% (w/v) agarose gel containing SYBR Safe (NEB) and visualized using UV transillumination. A 1 kb DNA ladder (NEB) was used as an indicator of size (0.5–10 kb).

### Cloning, expression and purification of Gcl, Hyi and GlxR

*E. coli* Rosetta cells containing pRARE-2 and either pET-19m(GlxR), pET-19m(Hyi) or pET-19m(Gcl) were grown in 2 l of LB containing 30 µg ml^−1^ chloramphenicol and 50 µg ml^−1^ carbenicillin. For the expression of Gcl, the cultures also contained 10 µg ml^−1^ thiamine, 10 µg ml^−1^ flavin, 0.66 M sorbitol and 2.5 mM betaine. For all cultures, at OD_600_ 0.5, the temperature was decreased to 20 °C and protein expression was induced by the addition of 1 mM isopropyl *β*-D-1-thiogalactopyranoside. After overnight growth, cells were harvested by centrifugation (12,000 ***g***, 10 min, 4 °C) and washed once in PBS. The cell pellets were resuspended in 50 ml lysis buffer (50 mM Tris pH 7.5, 400 mM NaCl, 10 mM imidazole and 5% (v/v) glycerol) containing one cOmplete™ Mini EDTA-free Protease Inhibitor Cocktail tablet (Roche). The cells were lysed by sonication on ice, and the sample was clarified by sedimentation (27,000 ***g***, 15 min, 4 °C). The cell-free supernatant was loaded onto an Ni-NTA agarose (Qiagen) column pre-equilibrated with lysis buffer. After washing overnight (~500 column volumes) with lysis buffer, the bound protein was eluted in 50 mM Tris, 400 mM NaCl, 300 mM imidazole and 5% (v/v) glycerol (pH 7.5). The sample was then dialysed overnight at 4 °C against 20 mM Tris, 100 mM NaCl, 5% (v/v) glycerol, 0.1 mM EDTA and 1 mM DTT (pH 7.5). For Gcl, His_6_-tagged TEV protease was added to the samples (1:50 mg TEV:mg protein) before dialysis to ensure cleavage of the N-terminal His_6_ tag on each of the over-expressed proteins. After dialysis, the solution was incubated on ice (30 min, gentle shaking) with a slurry of Ni-NTA agarose to remove the His_6_-TEV, cleaved His_6_ tags and any uncleaved target protein. The Ni-NTA beads were pelleted (3,050 ***g***, 30 min, 4 °C) and the protein supernatant was decanted. All protein samples were concentrated by ultrafiltration (VivaSpin column, 30,000 MWCO), then aliquoted and flash-frozen in liquid nitrogen. Protein concentration was determined by spectrophotometry using calculated molar extinction coefficients: Gcl, 59,870 M^−1^ cm^−1^; Hyi, 31,750 M^−1^ cm^−1^; and GlxR, 8,490 M^−1^ cm^−1^.

### Kinetic assays

All stocks for kinetic reactions were made up in dH_2_O, aliquoted and stored at −20 °C, except NADH which was made up at 10 mM in 0.01 M NaOH and stored at −80 °C. Protein aliquots were defrosted and diluted in dialysis buffer immediately before use. All reactions were carried out at 37 °C, and reaction progress was monitored through the decline of NADH absorbance at 340 nm using an Eppendorf BioSpectrophotometer with Eppendorf Uvette cuvettes. NADH concentrations were calculated using the molar extinction coefficient of 6.22 mM^−1^ cm^−1^ at 340 nm [25]. All experiments were carried out in triplicate unless noted, and GraphPad Prism v9 was used to analyse the data and extract kinetic constants.

GlxR activity with hydroxypyruvate as a substrate was measured in 500 µl reactions containing 20 mM MOPS, 0.2 mM NADH and 0–125 mM hydroxypyruvate (pH 7.5). Reactions were initiated by the addition of purified GlxR to a final concentration of 0.4 μg ml^−1^. Where indicated, Hyi was also added (to 10 µg ml^−1^ concentration). Kinetic constants were calculated using an allosteric sigmoidal model with respect to hydroxypyruvate concentration. Experiments were not carried out to determine kinetic constants with respect to NADH concentration as the methodology required a fixed initial NADH concentration. Rate constants were calculated based on the concentration of monomeric enzyme; for these purposes, and based on the AUC analyses, GlxR was assumed to be a tetramer in solution (Fig. S5).

GlxR activity with glyoxylate carboligase-derived TSA as a substrate was measured using a coupled assay. Briefly, an excess amount (10 µg) of Gcl was used to catalyse the conversion of glyoxylate into TSA. A limiting amount (0.2 µg) of GlxR was then used to convert the resulting TSA/hydroxypyruvate into glycerate, with concomitant oxidation of NADH. Reaction mixtures (500 µl) contained 20 mM MOPS, 0.2 mM NADH, 0–10 mM glyoxylate and, where required, 20 μg ml^−1^ of Hyi. The reactions were initiated by the addition of Gcl. Initial velocity was calculated through linear regression over the first part of the reaction, which varied from 15 s to 30 min, depending on the observed rate of NADH consumption, and kinetic constants were calculated using the Michaelis–Menten model. All experiments were carried out in triplicate. Rate constants etc. were calculated based on the concentration of monomeric enzyme; for these purposes, and due to the ambiguity in Gcl oligomeric status from the AUC experiments, native Gcl was assumed to be a tetramer, as reported for other glyoxylate carboligase enzymes [26]. For testing the impact of cofactors (Fig. S6), reactions were also supplemented with the indicated concentrations of thiamine pyrophosphate (TPP), flavin adenine dinucleotide (FAD) or MgCl_2_.

### Nuclear Magnetic Resonance (NMR) spectroscopy

Samples were prepared by dissolving the substrate (glyoxylate or hydroxypyruvate, as indicated) in buffered D_2_O (sodium phosphate, 50 mM) supplemented with 1 mM MgCl_2_, and the pH (pD) was adjusted to 7.0 using NaOD or DCl, as necessary. When enzymes were added, we used a 1:100 dilution (i.e. 7.5 µl of enzyme stock in 750 µl of buffered substrate). The mixture was gently stirred and the spectrum at *t*=0 h was measured. The sample was incubated at 37 °C for the indicated period of time, at which point subsequent NMR measurements were taken. The magnetic resonance spectra were recorded at 298 K using an internal deuterium lock on a Bruker Avance III 500(500 MHz, BBFO Smart probe) spectrometer. Proton chemical shifts are quoted in p.p.m. to the nearest 0.01 p.p.m. and are referenced to the residual non-deuterated solvent peak (D_2_O: 4.79). Proton assignments are supported by DEPT135, ^1^H-^13^C HSQC and ^1^H-^13^C HMBC spectra.

### Protein crystallization

For crystallization of GlxR, the over-expressed protein was then purified essentially as outlined above using Ni-NTA chromatography, except that the elution buffer was PBS, 0.1 M NaCl and 0.3 M imidazole. The purified protein was dialysed against 2×1 l PBS, 0.1 M NaCl and 1 mM DTT and concentrated to ~20 mg ml^−1^ by ultrafiltration (VivaSpin column, 30,000 MWCO). Protein purity was evaluated by SDS-PAGE and concentration was determined by UV-Vis spectrophotometry. For crystallization, a 50:50 mixture of protein solution and crystallization buffer was used, and the drops were developed using vapour diffusion. The crystallization buffers that yielded diffracting crystals of the *α* and *β* forms are given in Table S3. The crystal structures were solved essentially as described in [27].

## Results

### Bioinformatic analysis of the *gcl* cluster ORFs

Based on previous work where we characterized the structure, allosteric regulation and biochemical activity of PykF (PA1498) [7], we were drawn to investigate the function of the associated adjacent ORFs. PA1499 was predicted to encode a protein belonging to the glycerate kinase sub-group represented by TM1585 from *Thermotoga maritima*. This sub-group of enzymes exhibits only low sequence similarity with the better-characterized glycerate kinases associated with most *Firmicutes* and *Gammaproteobacteria*. Consequently, PA1499 shares only 19% and 21% identity (respectively) with the *E. coli* glycerate kinases, GlxK and GarK. PA1499 was also predicted to be a potential hydroxypyruvate reductase (Hpr), capable of converting hydroxypyruvate directly into glycerate (with concomitant oxidation of NADH). However, *P. aeruginosa* encodes a dedicated Hpr elsewhere on the genome (PA4626, *hprA*) and the most likely function of PA1499 is to phosphorylate glycerate. The next ORFs in the cluster, PA1500 and PA1501, were predicted to encode a TSA reductase (GlxR) and a hydroxypyruvate isomerase (Hyi), respectively, sharing 62% (GlxR) and 58% (Hyi) identity with the corresponding *E. coli* homologues. Finally, PA1502 was predicted to encode a glyoxylate carboligase (Gcl), 74% identical to the *E. coli* homologue. On the basis of these predictions, the uncharacterized genes are hereafter denoted *glxK* (PA1499), *glxR* (PA1500), *hyi* (PA1501) and *gcl* (PA1502), and we designate these ORFs collectively as the ‘*gcl* cluster’.

### Mutants defective in the *gcl* cluster cannot grow on allantoin as a sole carbon source

We predicted that the inactivation of ORFs in the *gcl* cluster would impair the ability of *P. aeruginosa* to grow on allantoin as a sole carbon source. To investigate this further, mutants containing transposon insertions in *glxR*, *hyi* and *gcl* were obtained from the UWGC two-allele library [23]. A full list of the strains and plasmids used in the current study is shown in Table S1. A *glxK*::Tn mutant was not available. All Tn insertions were confirmed by PCR amplifying across the junction between the disrupted ORF and the Tn, as recommended by the UWGC providers.

**Fig. 2.**
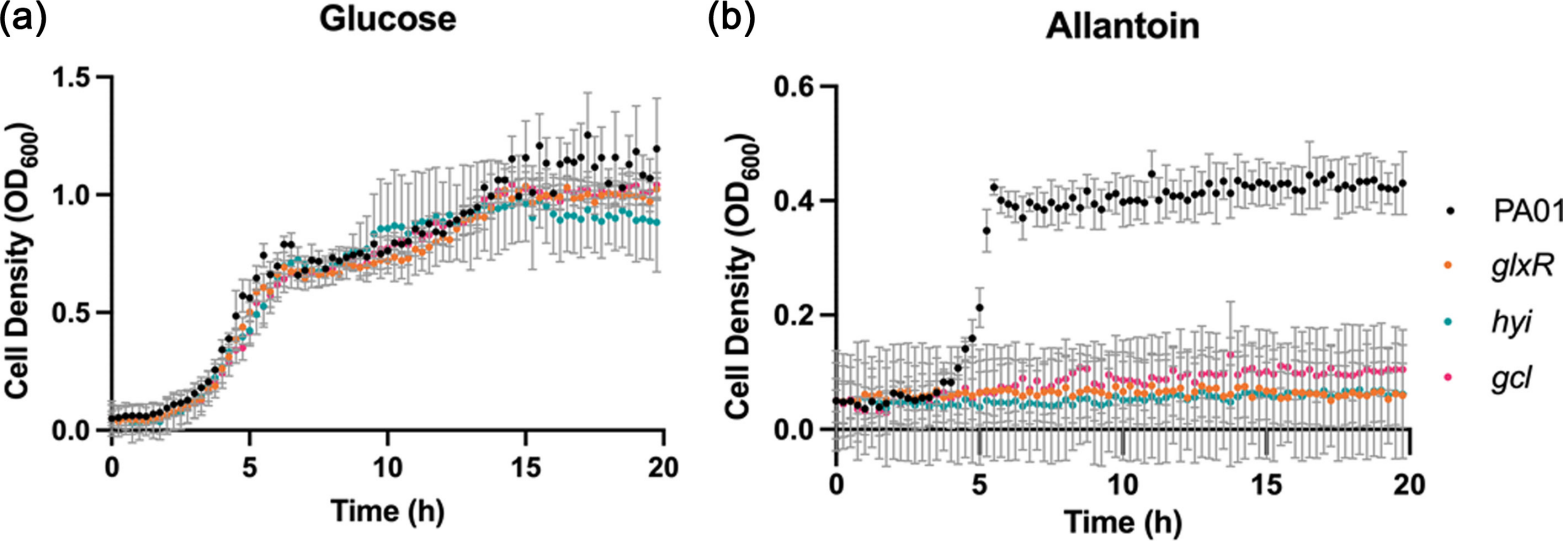
Disruption of ORFs in the *gcl* cluster impairs growth on allantoin as a sole carbon source. The figure shows growth (monitored as OD at 600 nm) of the progenitor strain (PA01) (black dots) and *glxR*::Tn (orange dots), *hyi*::Tn (blue/cyan dots) and *gcl*::Tn (pink/magenta dots) mutants in M9 minimal media supplemented with (**a**) 0.25% (w/v) glucose or (**b**) 0.33% (w/v) allantoin. The data represent the mean and sd of three biological replicates.

Each mutant and the progenitor (PA01) were grown in M9 minimal media supplemented with either glucose or allantoin as a sole carbon source. The WT grew on both carbon sources, indicating that *P. aeruginosa* can readily utilize allantoin (Fig. 2). However, whereas the *gcl* cluster mutants grew comparably to the WT on glucose, they failed to grow on allantoin. Interestingly, the pyruvate kinase in the proposed catabolic pathway (Fig. 1c) is not essential since we previously showed that a *pykA pykF* double mutant is able to grow, albeit at a reduced rate, on allantoin as a sole carbon source [7]. This indicates that the conversion of allantoin-derived phosphoenolpyruvate to pyruvate is not an essential step, presumably because the 2-phosphoglycerate produced in the GlxK-catalysed reaction can itself feed directly into the gluconeogenic pathway [18,28, 29].

### Allantoin induces expression from the *gcl* promoter

**Fig. 3.**
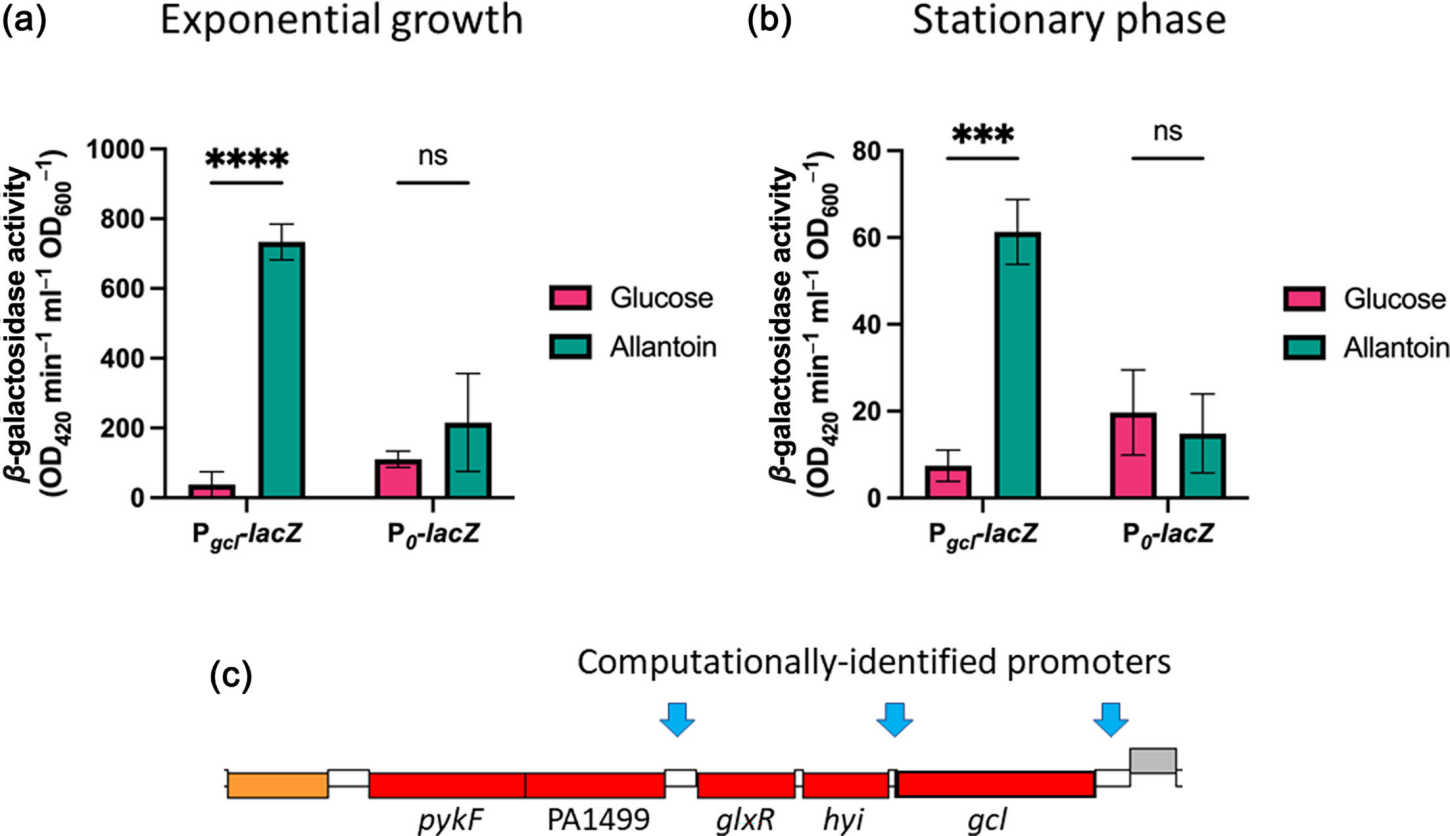
Expression from the *gcl* promoter is induced by allantoin. *β*-Galactosidase activity in cultures of PAO1 containing chromosomally integrated P*_gcl_-lacZ* (or, as a control, chromosomally integrated empty miniCTX-*lacZ* vector; P_0_*-lacZ*) harvested at (**a**) mid-log phase growth or (**b**) stationary phase. The growth medium was M9 medium supplemented with 0.33% (w/v) allantoin (green bars) or 0.25% (w/v) glucose (pink bars). *β*-Galactosidase activity is expressed in OD_420_ min^−1^ ml^−1^ OD_600_^−1^ (Miller units), and the data represent the mean and sd of three biological replicates. Significance was calculated using unpaired t-tests and is indicated by **** (*P*≤0.0001) and *** (*P*≤0.001). (**c**) Computationally predicted operon structure of the *gcl* cluster.

The GntR-family regulator, UgmR (PA1520), has been previously shown to bind upstream of both the *gcl* ORF (PA1502; Fig. 1c) and the allantoin catabolic gene cluster (PA1513-PA1518; Fig. 1a), thereby repressing the expression of the downstream ORFs. Indeed, the expression of all of the ORFs in the *gcl* cluster has been previously shown to be up-regulated in a △*ugmR* mutant [13]. This UgmR-mediated repression is relieved by uric acid, but not by allantoin [7,13]. However, PykF expression has been previously shown to be strongly induced in the presence of allantoin, suggesting that the expression of the *gcl* cluster may also be induced via a UgmR-independent route [7]. To investigate this further, we generated a chromosomally integrated miniCTX-based construct in which the *gcl* promoter (P*_gcl_*) was fused upstream of a promoter-less *lacZ*. MiniCTX-based constructs stably integrate at a neutral site (*attB*) in the chromosome of *P. aeruginosa* [24,30]. *β*-Galactosidase activity was then measured during growth on glucose or allantoin as a sole carbon source (Fig. S1). The expression from P*_gcl_* was significantly greater in cultures grown on allantoin compared with those grown on glucose in both the mid-log (*P*<0.0001) and stationary (*P*=0.0004) phase of growth (Fig. 3a, b). We also note that the expression from the P*_gcl_* promoter was tenfold greater in mid-log phase cultures compared with stationary phase cultures, as expected for enzymes involved in primary metabolism.

Although the *gcl* cluster ORFs are all up-regulated in a △*ugmR* mutant [13], computational analyses suggest that the cluster potentially comprises three transcriptional units (Fig. 3c), encoding *gcl* alone, *hyi* and *glxR* and *glxK* and *pykF*, respectively. To investigate this possibility further, we purified RNA from allantoin-grown cells and used PCR to assess whether ORFs in the derived cDNA are indeed assembled into these transcriptional units (Fig. S2). These analyses confirmed that *gcl* (Fig. S2E) and *glxR-hyi* (Fig. S2D) are indeed expressed as separate transcriptional units, although we failed to establish that *glxK* and *pykF* are bicistronic (Fig. S2C). However, this may simply indicate that the *glxK-pykF* transcript is present at only very low levels. Consistent with this, PCR amplification of *glxK* alone from the cDNA yielded a product with very low yield (Fig. S2A). PCR amplification across the boundary between *glxK* and *glxR*/*hyi* failed to yield a product from the cDNA template, although a product of the correct anticipated size was obtained with gDNA as a template (Fig. S2B). This is consistent with *glxK* and *glxR*/*hyi* being in separate transcriptional units. Similarly, PCR amplification across the boundary between *gcl* and *hyi* yielded only a very faint band from the cDNA [*cf*. a robust band from the gDNA template (Fig. S2C)]. This suggests that *gcl* and *hyi* can indeed be transcribed on a single transcript but that the expression level of that transcript is likely very low *cf*. the expression levels of the *gcl* and *hyi-glxR* transcripts. Taken together, our data favour the computationally predicted operon structure, with the caveat that there may be some run-through between *gcl* and *hyi-glxR* and that transcript levels of the *glxK-pykF* operon are low.

To investigate further how the *gcl* cluster operons might be regulated, we interrogated the data from a recent SELEX-based genome-wide identification of transcription factor binding sites in *P. aeruginosa* [31]. We considered transcription factors in this dataset to be potentially regulatory if they bound in a window spanning +100 to −400 nt relative to the start codon of the first ORF (*gcl*, *hyi* and *glxK*, respectively) in each of the predicted transcriptional units. For *gcl*, this approach confirmed the presence of a UgmR binding motif and also revealed binding sites for an uncharacterized GntR-family transcriptional regulator (PA0120) and an AraC-family transcriptional regulator (PA3782). For *hyi*, a binding site for a TetR-family repressor (PA1315) was identified, whereas for the *glxK-pykF* transcriptional unit, we identified a second binding site for PA3782, as well as a binding site for a global virulence/lifestyle regulator, AmrZ (PA3385) [32]. In summary, the transcriptional units in the *gcl* cluster are likely subject to regulation by several – sometimes overlapping – factors.

### *In vitro* biochemical activities of selected *gcl* cluster-encoded enzymes

**Fig. 4.**
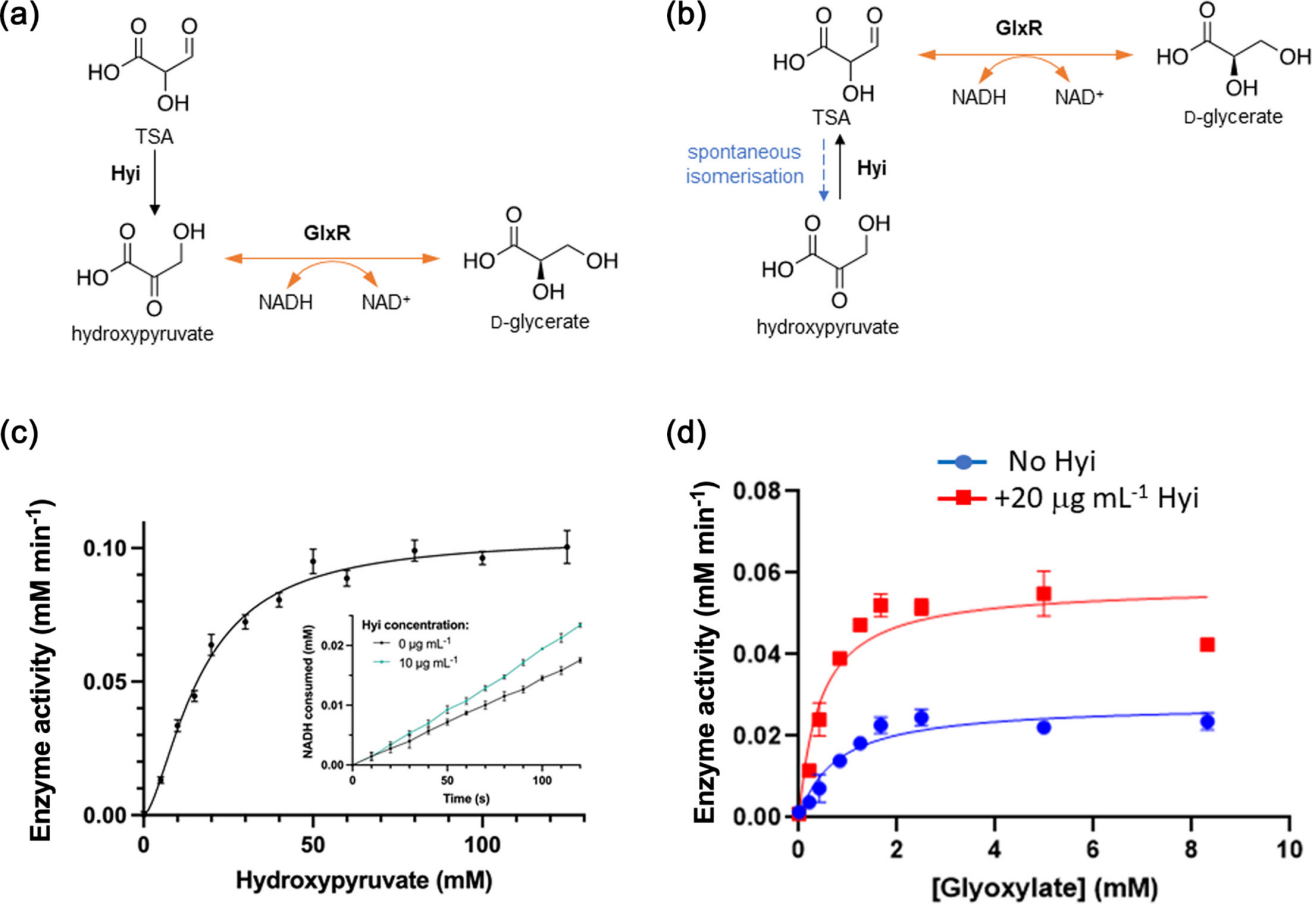
Reactions catalysed by GlxR and Hyi. Gcl catalyses the dimerization of two molecules of glyoxylate to form TSA. This TSA has two possible fates. (**a**) Hyi catalyses the conversion of TSA into hydroxypyruvate, which is then converted by GlxR into d-glycerate. (**b**) GlxR reduces TSA directly to yield d-glycerate. However, in a competing reaction, TSA also spontaneously tautomerizes to yield hydroxypyruvate. In this scenario, Hyi catalyses the conversion of hydroxypyruvate to TSA, thereby restoring TSA levels and increasing the rate of the GlxR-catalysed reaction. (**c**) GlxR-catalysed conversion of hydroxypyruvate into d-glycerate. The concentration of GlxR was 0.4 µg ml^−1^. Inset: Impact of Hyi addition on GlxR-catalysed reaction rate in the presence of 20 mM hydroxypyruvate. (**d**) The GlxR-catalysed conversion of *de novo* synthesized TSA from the Gcl-catalysed reaction into d-glycerate is increased in the presence of Hyi. Gcl was present at 20 μg ml^−1^ and GlxR at 0.4 μg ml^−1^.

Hydroxypyruvate isomerase (Hyi) has been previously described to catalyse the conversion of TSA into hydroxypyruvate, with the latter then being converted to glycerate by GlxR [18] (Fig. 4a). However, GlxR has also been proposed to catalyse the conversion of TSA directly into glycerate, which makes the Hyi-catalysed TSA ↔ hydroxypyruvate interconversion seem rather pointless. Mitigating, we note that TSA has been shown to readily tautomerize at neutral pH to yield hydroxypyruvate, as well as potentially undergoing other detrimental side reactions such as decarboxylation and oxidation [33,35]. This raises the question of whether the main function of Hyi might be to ensure that spontaneously generated hydroxypyruvate is immediately converted back to TSA, thereby minimizing the impact of these non-enzymatic side reactions (Fig. 4b). To investigate this further, we examined

1. Whether GlxR can use hydroxypyruvate as a substrate.
2. The kinetics of NADH oxidation by GlxR with *de novo* synthesized TSA as a substrate (derived directly from the Gcl-catalysed reaction).
3. Whether the inclusion of purified Hyi into the GlxR-catalysed reaction increases the rate of the reaction in the presence of Gcl-derived TSA.

The ORFs encoding Gcl, GlxR and Hyi were tagged with an N-terminal His_6_ tag and over-expressed (separately) in *E. coli*. The proteins were purified using Ni-NTA chromatography. The oligomeric state of each purified protein was determined using analytical ultracentrifugation-sedimentation velocity analyses (AUC-SV) (Figs S3–S5). *P. aeruginosa* GlxR was a tetramer in solution, whereas Hyi formed a dimer. The AUC-SV data for Gcl were ambiguous and potentially suggestive of the presence of mixed oligomeric forms and/or protein aggregation. *E. coli* Gcl has been previously reported to exist in tetrameric and dimeric forms [22,26, 36]. Purified *P. aeruginosa* Gcl was yellow in colour (*λ*_max_ 445 nm), commensurate with it containing a bound FAD moiety [37].

In addition to the FAD moiety, Gcl from *E. coli* (and presumably, also Gcl from *P. aeruginosa*) contains a bound TPP cofactor. Given that some of the Gcl-associated FAD and TPP may have potentially been lost during the purification washing steps, we first tested whether Gcl activity was increased when the assay mixtures were supplemented with 100 µM FAD and/or 10 µM TPP. TPP addition had little impact, but FAD supplementation roughly doubled the enzyme activity (Fig. S6A). Mg^2+^ was found to be essential for optimal activity in the coupled reactions, and GlxR was found to have a ~1.8-fold preference for NADH as a substrate compared with NADPH (Fig. S6A,B).

The rate of NADH oxidation was used to measure GlxR activity (i) using hydroxypyruvate as a substrate (Fig. 4c) or (ii) in a coupled reaction using TSA synthesized *de novo* by Gcl as a substrate (Fig. 4d). No NADH was oxidized in the absence of GlxR (Fig. S6C), and the rate of the reaction increased proportionally with the amount of GlxR added (Fig. S6D). With commercially available hydroxypyruvate as a substrate, GlxR kinetics were best fit using a sigmoidal (Hill) model, with *h*=1.6±0.2, indicating some positive cooperativity between the active sites in the GlxR tetramer. This sigmoidal kinetic behaviour of the enzyme meant that *K*_half_ had to be used in place of *K*_M_ to describe the kinetic parameters. *K*_half_ was 16.7 mM and *k*_cat_ was 33.5 s^−1^, yielding a *k*_cat_/*K*_half_ value of 2.0 s^−1^ mM^−1^. Superficially, these data could indicate that hydroxypyruvate is indeed a GlxR substrate, albeit a very poor one. However, it must be borne in mind that hydroxypyruvate and TSA are presumably in equilibrium in solution, so some TSA may also be present in this reaction mixture. If this is correct, and assuming that Hyi catalyses the interconversion of hydroxypyruvate ↔ TSA, then the rate of this GlxR-catalysed reaction should increase in the presence of Hyi. It did, but only slightly (Fig. 4c, inset). We next examined whether GlxR shows any activity against TSA synthesized *de novo* in the Gcl-catalysed reaction. It did (Fig. 4d) and did so in proportion to the amount of Gcl present (Fig. S6E,F). In the presence of Gcl-derived TSA, the reactions manifested Michaelis–Menten kinetics and could be described using *K*_M_ instead of *K*_half_. The *K*_M_ was 0.8 mM and *k*_cat_ was 34.7 s^−1^, yielding a *k*_cat_/*K*_M_ value of 45.8 s^−1^ mM^−1^, substantially greater than the 2.0 s^−1^ mM^−1^ obtained with hydroxypyruvate as a directly supplied substrate. Moreover, in the presence of Hyi, *k*_cat_/*K*_M_ increased to 163.5 s^−1^ mM^−1^ [*K*_M_ was 0.4 mM and *k*_cat_ was 70.6 s^−1^ (Fig. 4d)]. This is consistent with the notion that Hyi counters spontaneous TSA loss *in vitro* by catalysing the conversion of hydroxypyruvate back to TSA (as in Fig. 4b). We note that the true value of *k*_cat_/*K*_M_ is likely even higher than 163.5 s^−1^ mM^−1^, since even in the presence of Hyi, some TSA will inevitably tautomerize to yield hydroxypyruvate, lowering the overall concentration of the substrate.

**Fig. 5.**
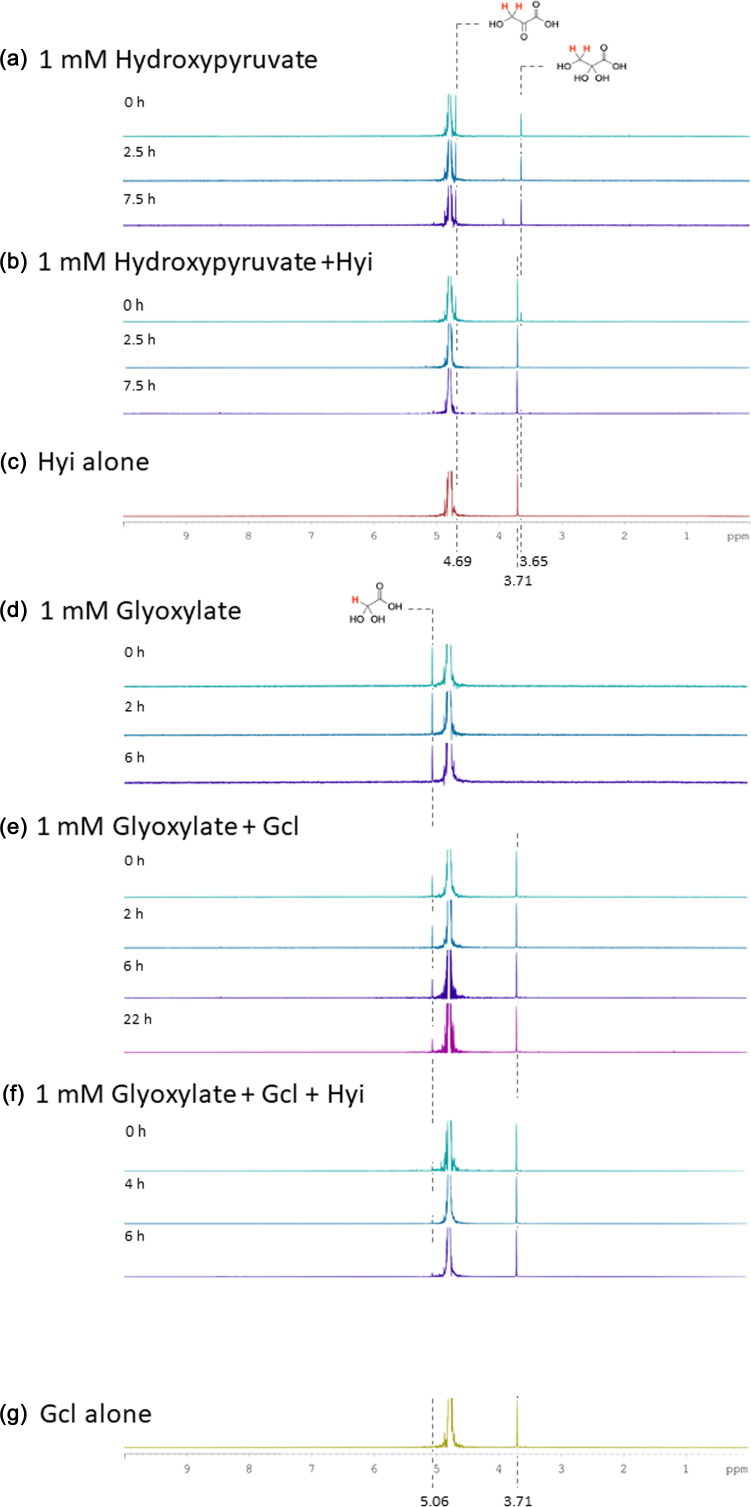
The figure shows [^1^H]-NMR spectra for the indicated compounds in the presence of the indicated enzymes, incubated for the indicated periods of time at 37 °C in D_2_O. (**a**) Spectrum of hydroxypyruvate on its own. (**b**) Spectrum of hydroxypyruvate in the presence of Hyi, which resulted in an increased rate of disappearance of the hydroxypyruvate signals (3.65 and 4.69 p.p.m.), likely due to continuous isomerization and exchange of hydrogens for deuterium atoms. (**c**) Control NMR of an aliquot of Hyi (in the absence of hydroxypyruvate) in D_2_O, indicating that the peak at 3.71 p.p.m. is derived from the enzyme preparation. (**d**) Spectrum of glyoxylate on its own. (**e**) Spectrum of glyoxylate in the presence of Gcl. Note the slow disappearance of the glyoxylate peak at 5.06 p.p.m. (**f**) Spectrum of glyoxylate in the presence of Gcl and Hyi, which resulted in rapid disappearance of the glyoxylate signal. (**g**) Control NMR of an aliquot of Gcl in D_2_O, again indicating that the peak at 3.71 p.p.m. is derived from the enzyme preparation.

To further investigate the likely function of Hyi, we used ^1^H-NMR. Hydroxypyruvate yielded stable peaks at 4.69 and 3.65 p.p.m. (for the hydrate) (Fig. 5a). In the presence of Hyi, both hydroxypyruvate peaks had lessened in intensity by the time the first measurement could be made (‘*t*=0 h’) and had disappeared by the time of the second measurement (*t*=2.5 h) (Fig. 5b). We also noticed a new peak appear at 3.71 p.p.m. on the addition of Hyi. However, this peak also appeared when Hyi alone (Fig. 5c) or Gcl alone (Fig. 5g) was examined, indicating that it is likely derived from the enzyme preparation, rather than being a product of the reaction. In this regard, in the reaction mixtures containing Hyi and hydroxypyruvate, we did not see any new peaks appear. One explanation for this is that such peaks may be present but are masked by the residual water signal (4.75–4.90 p.p.m.). In the presence of glyoxylate alone, we noted a stable signal from the hydrate of the aldehyde at 4.06 p.p.m. (Fig. 5d). In the presence of Gcl, this hydrated glyoxylate peak slowly diminished in intensity over a 22-h period (Fig. 5e), but with no new peak(s) appearing except for the enzyme preparation-derived peak at 3.71 p.p.m. Presumably, the slow rate of the Gcl-catalysed reaction in this assay reflects the lack of any coupled reaction to ‘pull’ the equilibrium further over towards the reaction product(s). Consistent with this (and with the kinetic data in Fig. 4), in the presence of Hyi, the Gcl-catalysed reaction was much faster; the hydrated glyoxylate peak disappeared before the first measurement could be taken (Fig. 5f). There are two possible explanations for this enhancement of the Gcl rate. First, Hyi is not a hydroxypyruvate isomerase but, instead, converts the hydrated glyoxylate into a better substrate for Gcl. Second, Hyi converts the product of the Gcl reaction (presumably, TSA) into something else (not hydroxypyruvate; we observed neither the *keto* form of this product nor its hydrate in Fig. 5f), thereby pulling the equilibrium over to the right-hand side. Irrespective of which interpretation is correct, we conclude that Hyi accelerates the rate of the Gcl-catalysed reaction.

### GlxR is a conformationally flexible enzyme

As part of our characterization of the *gcl* cluster enzymes, we also tried crystallizing Hyi and GlxR with the goal of solving their X-ray crystal structures. Hyi failed to yield diffraction-quality crystals, but GlxR yielded crystals in two different conditions. Only three structures for TSA reductases have been previously reported, and only one of these is associated with an accompanying description in the published literature [38]. Two of these TSA reductases are from *Salmonella enterica* Typhimurium; GarR_Sty_ (1VPD, [38]) and GlxR_Sty_ (PDB 1YB4, unpublished), which share 43% and 61% (respectively) amino acid identity with GlxR from *P. aeruginosa*. The third structure is associated with an uncharacterized TSA reductase from *Polaromonas* sp. JS666 (PDB 4DLL, unpublished).

**Fig. 6.**
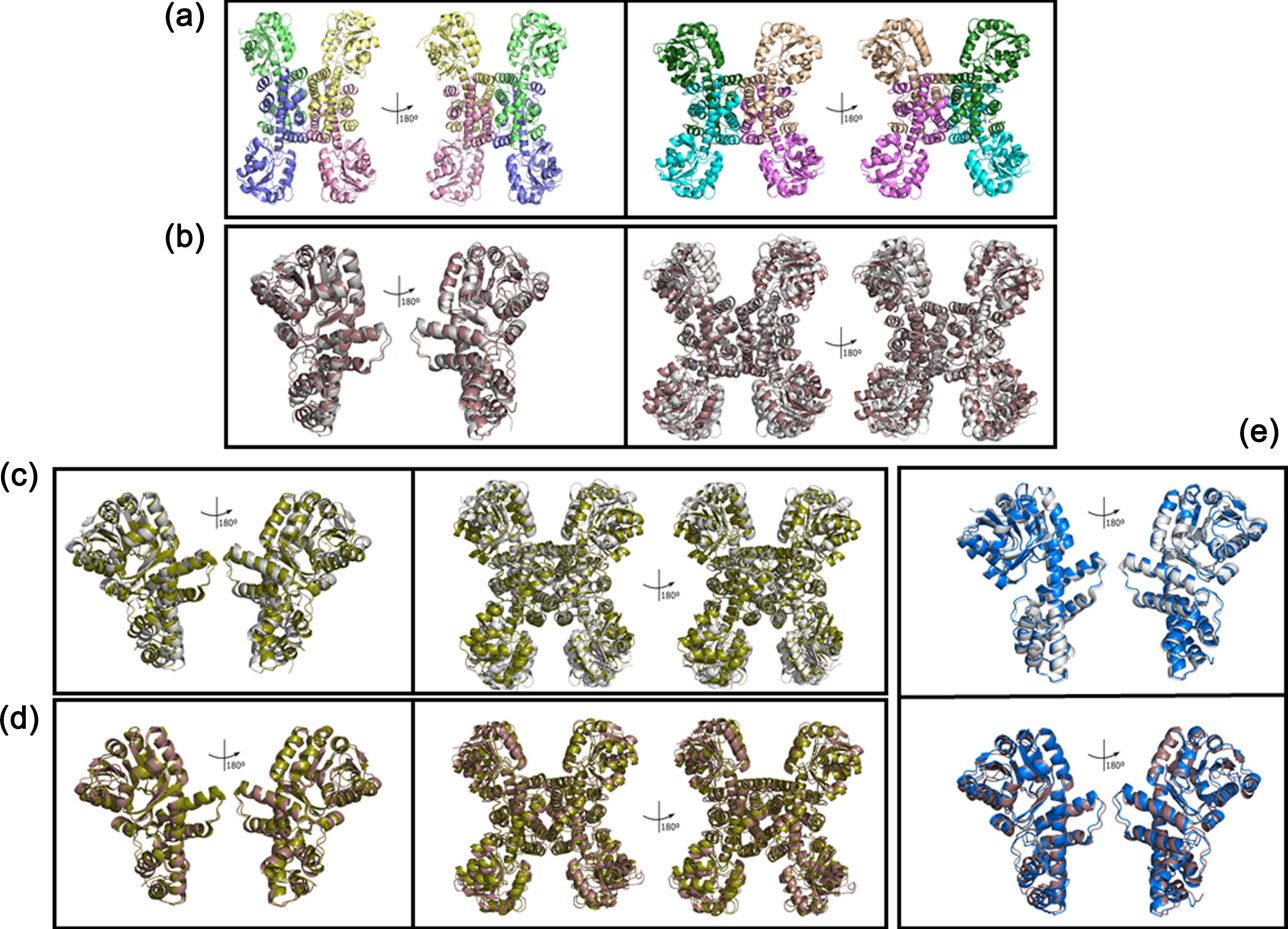
Structures of GlxR from *P. aeruginosa*. (**a**) X-ray crystal structures of GlxR *α* (left panel) and *β* (right panel) forms. The protomers in each structure are represented in different colours, and the tetramers are rotated through 180°, as indicated. (**b**) Protomers (left panel) and tetramers (right panel) of the *α* (grey) and *β* (brown) forms of GlxR superimposed. The root mean square deviation (RMSD) for superposition of the promoters is 2.24 Å and for superposition of the tetramers is 3.60 Å. (**c**) Protomers (left panel) and tetramers (right panel) of the *α* form of GlxR (grey) superimposed on the structure of GarR_Sty_ (1VPD, green). The RMSD for superposition of the protomers is 3.02 Å and for superposition of the tetramers is 5.82 Å. (**d**) Protomers (left panel) and tetramers (right panel) of the *β* form of GlxR (brown) superimposed on the structure of GarR_Sty_ (1VPD, green). The RMSD for superposition of the protomers is 1.68 Å and for superposition of the tetramers is 3.34 Å. (**e**) Protomer of the GlxR *α* conformer (upper panel, grey) and the GlxR *β* conformer (lower panel, brown) superimposed on the structure of GlxR predicted by AlphaFold (AF-Q9I3L2, blue). The RMSD for superposition of the *α* conformer is 1.30 Å and for superposition of the *β* conformer is 1.57 Å.

**Fig. 7.**
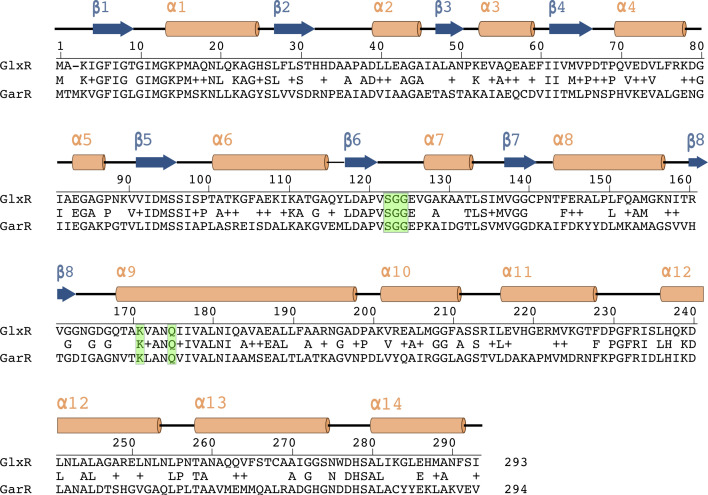
Sequence alignment of GlxR from *P. aeruginosa* and GarR from *S. enterica* Typhimurium. Secondary structural elements derived from the X-ray crystal structure of GlxR are indicated, as are the conserved active site residues (boxed in green).

GlxR was crystallized from drops containing 0.1 M sodium acetate (pH 4.5) and 25% (w/v) polyethylene glycol smear medium and from drops containing 0.1 M Tris-HCl (pH 8.5), 20% (w/v) polyethylene glycol smear high and 20% glycerol. The resulting X-ray structures were designated the *α* and *β* forms, respectively. The structure of the *α* form was solved to 2.07 Å resolution and the structure of the *β* form was solved to 2.21 Å resolution (Fig. 6a, Table S3). Both conformers contain an N-terminal domain comprised of nine *α*-helices surrounding a core of nine *β*-strands, and an exclusively *α*-helical C-terminal domain. In GlxR_Sty_ and GarR_Sty_, the active site is located in the hinge region between the two domains, and the same active site residues are conserved in GlxR too (Fig. 7). The quaternary structure of the *α* form is a ‘squashed tetramer’ (Fig. 6a, left panel), consistent with the oligomeric configuration inferred from the AUC-SV data in Fig. S5. By contrast, the oligomeric arrangement of the *β* form tetramer adopts a more open, expanded structure (Fig. 6a, right panel). However, when we superimposed the individual *α* and *β* protomers, they had a very similar structure (Fig. 6b, left panel), indicating that the differences in quaternary structure are primarily due to rigid body rearrangements of the protomers. Given that inter-protomer contacts in both the *α* and *β* forms of the enzyme are mediated through interactions between the C-terminal domains, this rigid body movement can be thought of as a rocking motion around a C-terminal pivot point. Commensurate with this, when the tetrameric structures of the *α* and *β* conformers are superimposed, the N-terminal domains (i.e. the domains most distant from the pivot point) show the largest differences in spatial disposition (Fig. 6b, right panel).

The overall structure of each GlxR protomer was similar to that of GarR_Sty_, with GlxR*β* displaying a smaller root mean square deviation (RMSD) (1.68 Å) compared with the GlxR*α* conformer (RMSD 3.02 Å). GarR_Sty_ is the only other TSA reductase where the active site residues have been explicitly identified (Fig. 6c, d). These catalytic residues (S120, G121, G122, K171 and Q173) are conserved in GlxR, where they form a distinct pocket in the hinge region between the N- and C-terminal domains of the protein. Interestingly, and although the *α* conformer is more ‘compact’ than the *β* conformer, the distance between the serine –OH and the lysine –NH_3_^+^ was larger in GlxR*α* (5.3 Å) than it was in GlxR*β* (4.6 Å). For comparison, the same distance in GarR_Sty_ is 4.3 Å. Given that the GlxR*β* tetramer is also structurally more similar (*cf*. the GlxR*α* tetramer) to the GarR_Sty_ tetramer, these data suggest that the GlxR*β* conformer is likely to be more physiologically relevant. In this regard, it is noteworthy that when we compared the *α* and *β* conformer structures with the GlxR structure predicted by AlphaFold (AF-Q9I3L2), the latter was most similar to GlxR*α* form (Fig. 6e).

## Discussion

In this work, we elucidate the function of a cluster of ORFs that we have designated as ‘the *gcl* cluster’, PA1498–PA1502. Based on bioinformatic analyses and analogy with orthologues in other organisms (particularly, *P. putida* [18]), we predicted that PA1499 encodes a likely glycerate kinase (*glxK*), PA1500 a TSA reductase (*glxR*), PA1501 a hydroxypyruvate isomerase (*hyi*) and PA1502 a glyoxylate carboligase (*gcl*). Mutants carrying Tn insertions in *glxR, hyi* and *gcl* were unable to grow on allantoin as a sole carbon source. This may reflect an inability to generate carbon skeletons for growth, or the accumulation of a toxic pathway intermediate. The second possibility is supported by evidence from Cusa *et al*. [39], who demonstrated impaired growth of *E. coli* glycerate kinase mutants on xylose with allantoin as a sole nitrogen source; in that study, glyoxylate was postulated to be a toxic intermediate. However, in our hands, the *glxR*, *hyi* and *gcl* mutants were able to grow robustly in a medium containing glucose and allantoin (Fig. S1C). This suggests that if a toxic intermediate does exist, *P. aeruginosa* has other pathways capable of removing it. In light of this, it seems most likely that mutants in *glxR*, *hyi* and *gcl* are unable to grow on allantoin as a sole carbon source because they cannot generate carbon skeletons for growth. Of course, it is possible that not all of these ORFs are essential for growth on allantoin; whenever Tn insertions are being used, the issue of polarity needs to be acknowledged. However, the IS*phoA*/hah in the *gcl* mutant (PW3712) is inserted in the forward direction and contains an outward-reading constitutive promoter which should enable transcription of the downstream ORFs. Based on this, *gcl* is likely to be conditionally essential for growth on allantoin. By contrast, the IS*lacZ*/hah insertion in *hyi* and the IS*phoA*/hah insertion in *glxR* are both in the ‘reverse’ orientation, so in principle, polarity may well be an issue in these cases. Mitigating, we note that *hyi* and *glxR* form a transcriptional unit that is separate from that of the upstream *gcl* and the downstream *glxK*/*pykF* bicistron (Fig. 3c). In the light of this, and given that Tn insertions in both *hyi* and *glxR* prevent growth on allantoin, it seems likely that at least *glxR* is conditionally essential for growth on this substrate.

We previously demonstrated that PA1498 encodes a pyruvate kinase isozyme (PykF) [7], but the other ORFs in the *gcl* cluster remained uncharacterized. Here, we show that purified GlxR and Gcl do indeed exhibit TSA reductase and glyoxylate carboligase activity when assayed *in vitro* and that Hyi increases the rate of the Gcl-catalysed reaction. A formal possibility is that Hyi catalyses the interconversion of TSA and hydroxypyruvate, thereby offsetting losses of TSA (the product of the Gcl-catalysed reaction) due to spontaneous, non-enzymatic tautomerization. Consistent with this, the inclusion of Hyi into the Gcl-catalysed reaction mixtures increased the rate of glyoxylate conversion in both kinetic- and NMR-based assays. However, and on a note of caution, we failed to identify either a distinct TSA signal in the NMR or Gcl-catalysed conversion of glyoxylate into either the *keto* or the hydrated form of hydroxypyruvate, so it is possible that Hyi affects this reaction in ways other than that proposed here. Whatever its function, Hyi appears to play an important role in growth on allantoin.

We also present the X-ray crystal structure(s) of apo-GlxR, demonstrating that the enzyme can potentially adopt more than one distinct tetrameric configuration *in vitro* (Fig. 6). Which of these structures most closely approximates the physiologically relevant structure is not clear. However, and with more than one experimentally determined crystal structure in hand, we felt it would be instructive – especially given its widespread deployment in molecular biology – to compare the experimentally determined crystal structures of the GlxR*α* and GlxR*β* protomers with that of the AlphaFold prediction (Fig. 6e). The predicted (AlphaFold) structure was a slightly better fit with the GlxR*α* conformer. By contrast, the GlxR*β* conformer – at both the protomer and tetramer level of organization – was a better fit than GlxR*α* with the X-ray crystal structure of a GlxR homologue, GarR_Sty_. On balance, it seems likely that the GlxR*α* conformer may be a product of crystal packing constraints. Nevertheless, these data highlight the potential limitations imposed by the ‘training dataset’ in AlphaFold predictions and the ongoing need for experimental structure determinations.

Although the expression of the *gcl* cluster is strongly induced by allantoin, it is not clear which transcription factor(s) mediate this allantoin-dependent de-repression/activation. As noted in the Results section, UgmR (encoded by PA1520) is probably not involved, although a number of other transcriptional regulators (including PA0120, PA3782, PA1315 and PA3385) were also identified as having binding sites in the likely operator regions of the *gcl* cluster. Interestingly, one of these, the AraC family regulator PA3782, was inferred to bind upstream of both the *gcl* and *glxK*/*pykF* transcriptional units. PA3782 is of particular interest because it contains a PfpI-like domain. Given the recent identification of PfpI as a [methyl]glyoxalase in *P. aeruginosa* [40], and given the involvement of the *gcl* cluster in aldehyde metabolism, a parsimonious hypothesis is that the PfpI domain in PA3782 may be involved in sensing, e.g. glyoxylate or TSA accumulation in the cell. Current efforts are aimed at investigating these potential regulators and the allantoin-inducibility of the *gcl* cluster further.

Somewhat surprisingly (given its possession of a pathway for the conversion of glyoxylate to pyruvate), *P. aeruginosa* was unable to grow on 2-carbon substrates that likely feed into central metabolism via glyoxylate, such as ethylene glycol or glycolate (Fig. S1B). This may reflect the extremely low level of expression of the *gcl* cluster in the absence of allantoin (Fig. 3). However, the activity of *gcl* pathway enzymes has been previously detected in extracts of glycolate-grown *P. putida* and in extracts of other poorly characterized *Pseudomonas* species [14,17], so it seems likely that in some species/strains, 2-carbon substrates may also induce the expression of the *gcl* cluster. Consistent with this, differing induction of the *gcl* pathway has been shown between two strains of *P. putida* grown on glycolic acid, glyoxylate and ethylene glycol [29]. Nevertheless, it should also be borne in mind that, in itself, low-level enzyme activity may not necessarily be sufficient to enable growth on a substrate. For example, to enable *P. putida* KT2440 to grow on ethylene glycol as a sole carbon source, the expression of the orthologous *gcl* cluster needs to be artificially elevated from an inducible *P_tac_* promoter [18].

In summary, we show here that the ORFs PA1498–PA1502 encode a pathway for allantoin-derived glyoxylate metabolism in *P. aeruginosa*. The operon encoding these ORFs is strongly induced by allantoin, and mutation of the *gcl*, *hyi* and *glxR* ORFs prevents growth on allantoin as a sole carbon source. However, the work also raises some interesting questions for downstream research. First, how does allantoin derepress/activate expression of the *gcl* cluster? Several potential transcriptional regulators have been identified and discussed above, but another possibility is that the nearby gene encoded by PA1504 is involved. PA1504 was not included in the SELEX analysis reported in [31]. PA1504 encodes a TetR-type transcriptional regulator, with an N-terminal helix-turn-helix motif, followed by a YcdC/RutR-type C-terminal domain. This may be significant because RutR is known to negatively regulate transcription of the *rut* operon in *E. coli* (involved in pyrimidine utilization) [41]. This possibility is currently being investigated. Second, why does Hyi increase the rate of the Gcl-catalysed reaction? As noted elsewhere, although the kinetic and NMR data are consistent with Hyi doing this, there is still some ambiguity in exactly how it accomplishes this. Again, this is an area that we are actively investigating.

## Supplementary material

10.1099/mic.0.001635 Uncited Supplementary Material 1.

## Abbreviations

AUC-SV: analytical ultracentrifugation-sedimentation velocity
EDP: Entner–Doudoroff pathway
EMP: Embden–Meyerhof–Parnas
FAD: flavin adenine dinucleotide
gDNA: genomic DNA
TPP: thiamine pyrophosphate
TSA: tartronate semialdehyde
