# Supplementary material for: An allantoin-inducible glyoxylate utilization pathway in Pseudomonas aeruginosa
Source: Microbiology (Reading). 2025 Dec 10;171(12):001635. doi: 10.1099/mic.0.001635 (PMC12694930; doi:10.1099/mic.0.001635)
Supplement: Uncited Supplementary Material 1. [file mic-171-01635-s001.pdf]

Supplementary Material.

Table S1. List of bacterial strains and plasmids used in this study. Kan^R, kanamycin resistant; Cm^R, chloramphenicol resistant; Tet^R, tetracycline resistant; Cb^R, carbenicillin resistant.

**Bacterial strains**	**Description**	**Source**
PA01	Wild-type strain of *P. aeruginosa*	B. Iglewski (University of Rochester, USA)
SM10(λpir) miniCTX*lacZ*	*E. coli* strain harbouring miniCTX*lacZ* plasmid, Kan^R, Tet^R	(Becher & Schweizer, 2000)
JM109 miniCTX*lacZ*-P_{gcl}	*E. coli* strain harbouring miniCTX*lacZ*-P_{gcl}, Tet^R	This study
P_{gcl}-*lacZ*	PA01 containing *attB::*miniCTX*lacZ-*P_{gcl}, Tet^R	This study
P_0-*lacZ*	PA01 containing *attB::*miniCTX*lacZ*, Tet^R	This study
PW3707	PA01 harbouring transposon (IS*phoA*/hah) insertion in *glxR* at position 147/891 (PA1500::IS*phoA*/hah), Tet^R	UWGC mutant bank
PW3710	PA01 harbouring transposon (IS*lacZ*/hah) insertion in *hyi* at position 141/783 (PA1501::IS*lacZ*/hah), Tet^R	UWGC mutant bank
PW3712	PA01 harbouring transposon (IS*phoA*/hah) insertion in *gcl* at position 1429/1776 (PA1502::IS*phoA*/hah), Tet^R	UWGC mutant bank
**Plasmids**		
pET19m-(GlxR)	pET-19m containing the *glxR* ORF with an N-terminal His_6-tag and TEV cleavage site, Cm^R, Cb^R	This study
pET19m-(Hyi)	pET-19m containing the *hyi* ORF with an N-terminal His_6-tag and TEV cleavage site, Cm^R, Cb^R	This study
pET19m-(Gcl)	pET-19m containing the *gcl* ORF with an N-terminal His_6-tag and TEV cleavage site, Cm^R, Cb^R	This study

Table S2. List of primers used in this study.

Primers	Sequence	Purpose
P_{gcl}HindIIIF	cctaagcttCAGCCGCGACGACTTGCC	Amplification of *gcl* promotor, with HindIII recognition site
P_{gcl}EcoRIR	gctgaattcTTCTGGCCATCGTTTTGTTCCTCG	Amplification of *gcl* promotor, with EcoRI recognition site
mcl3997F	GCTGTTTCCTGTGTGATAAAG	Confirm insertion into miniCTX*lacZ*
mcl4255R	ATACGACTCACTATAGGGCG	Confirm insertion into miniCTX*lacZ*
P_{serup2}	CGAGTGGTTTAAGGCAACGGTCTTG	Confirm miniCTX*lacZ* integration into PA01 (chromosomal sequence). Adapted from Hoang *et al.* (2000).
$P_{serdown2}$	TTCGGCCTGGTGGAACAACTC	Confirm miniCTX*lacZ* integration into PA01 (chromosomal sequence). Adapted from Hoang *et al.* (2000).
mcl961	CCGGTCGCTACCATTACCAGTTG	Confirm miniCTX*lacZ* integration into PA01 (plasmid sequence).
mcl8810	GATTTCATACACGGTGCCTGAC	Confirm miniCTX*lacZ* integration into PA01 (plasmid sequence).
PA1502_for (gcl)	CCGACTCCATCCCGATTCTC	Forward primer for Q-RT-PCR (*gcl*)
PA1502_rev (gcl)	CGTTGATGGCGTTGATCTCG	Reverse primer for Q-RT-PCR (*gcl*)
PA1499_for (glxK)	CGAAGTGGTGGAGAAGCACT	Forward primer for Q-RT-PCR (*glxK*)
PA1499_rev (glxK)	GAAGTCGTTCACGTTGGTGC	Reverse primer for Q-RT-PCR (*glxK*)
PA1501_for (hyi)	TGTCCATGCTGTTCACCGAG	Forward primer for Q-RT-PCR (*hyi*)
PA1501_rev (hyi)	CCAGGTGCTCGAAGAGGAAG	Reverse primer for Q-RT-PCR (*hyi*)
PA1500_rev (glxR)	GAGTGGTCCCAGTTACTGCC	Reverse primer for Q-RT-PCR (*glxR*)
PA1498_rev (pykF)	TTGCTCGTTGACCACCGAAT	Reverse primer for Q-RT-PCR (*pykF*)

Table S3. Crystallographic statistics.

**Structure**	**A**	**B**
**PDB ID Code**	9RFA	9RB2
Isoform designation	α form	β form
**Data Collection**		
Wavelength (Å)	0.9763	0.9537
Resolution range (Å)	60.73 - 2.07 (2.100 - 2.070)	73.22 - 2.21 (2.250 - 2.210)
Space group	P 21 21 21	C 1 2 1
Unit cell		
a, b, c (Å)	80.44 121.47 132.84	79.18 109.2 146.69
a, b, g (°)	90.00 90.00 90.00	90.00 93.39 90.00
Total reflections	1091896 (52372)	441421 (22669)
Unique reflections	80069 (3916)	61556 (3061)
Multiplicity	13.6 (13.4)	7.2 (7.4)
Completeness (%)	100.0 (99.1)	98.7 (97.5)
Mean I/ sigma(I)	6.7 (0.3)	4.6 (0.7)
R-merge	0.218 (4.61)	0.446 (2.81)
R-pim	0.061 (1.30)	0.178 (1.10)
CC1/2	0.992 (0.40)	0.978 (0.35)
**Refinement**		
Resolution range (high resolution) (Å)	60.73 - 2.07 (2.09 - 2.07)	73.22 - 2.21 (2.23 - 2.21)
Reflections used in refinement	76426 (1529)	61442 (1229)
Reflections used for R-free	1975	2032
R-work	0.270 (0.456)	0.235 (0.377)
R-free	0.315 (0.497)	0.273 (0.417)
Number of total atoms	8758	9145
Macromolecules	8709	8784
Ligands	5	0
Solvent	44	361
RMS (bonds) (Å)	0.008	0.008
RMS (angles) (°)	1.04	0.98
RMS (dihedral angles) (°)	3.12	3.14
Average B-factor	58.9	39.5
**Crystallisation Conditions**	18% v/v PEGSH, 0.2 M $(NH_4)_2SO_4$, 0.1 M ADA, pH 6.5	25% v/v PEGSM, 0.1 M Na Acetate, pH 4.5

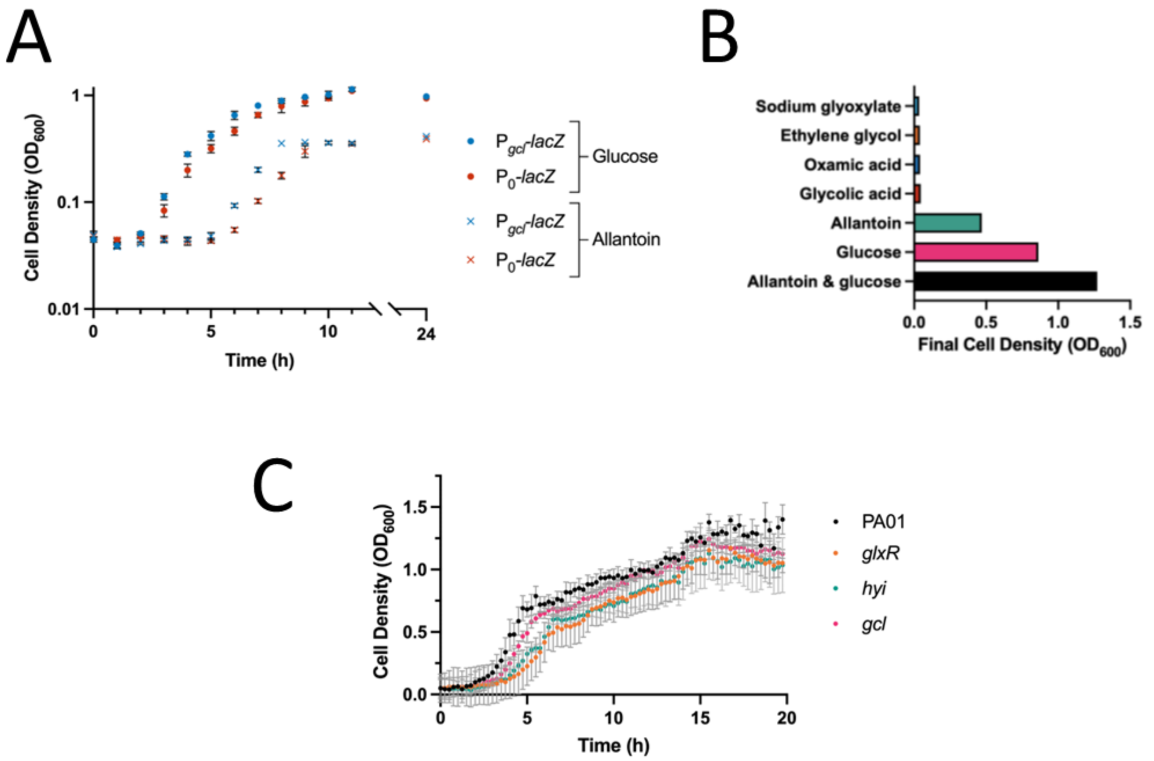

Figure S1. Growth rates of the PAO1::P_{gcl}-*lacZ* and PAO1::P_0-*lacZ* derivatives on glucose and allantoin, and growth of PAO1 on alternative C2 carbon sources. (A) PAO1 containing chromosomally-integrated P_{gcl}-*lacZ* and P_0-*lacZ* was grown in M9 minimal media supplemented with 0.25% (w/v) glucose or 0.33% (w/v) allantoin, and the optical density at 600 nm was measured at the indicated time points. The data represent the mean of three biological replicates ± SD. The data were plotted using GraphPad Prism. **(B)** *P. aeruginosa* (PAO1) cell density after 24 h growth on a variety of carbon sources. Carbon sources were added at final concentrations (w/v) of 0.5% sodium glyoxylate, 0.2% ethylene glycol, 0.4% oxamic acid, 0.3% glycolic acid, 0.33% allantoin and 0.25% glucose. The cultures were incubated at 37°C. Chart plotted with GraphPad Prism. **(C)** Growth of the indicated *gcl* cluster mutants in M9 minimal media containing 0.25% glucose and 0.33% allantoin.

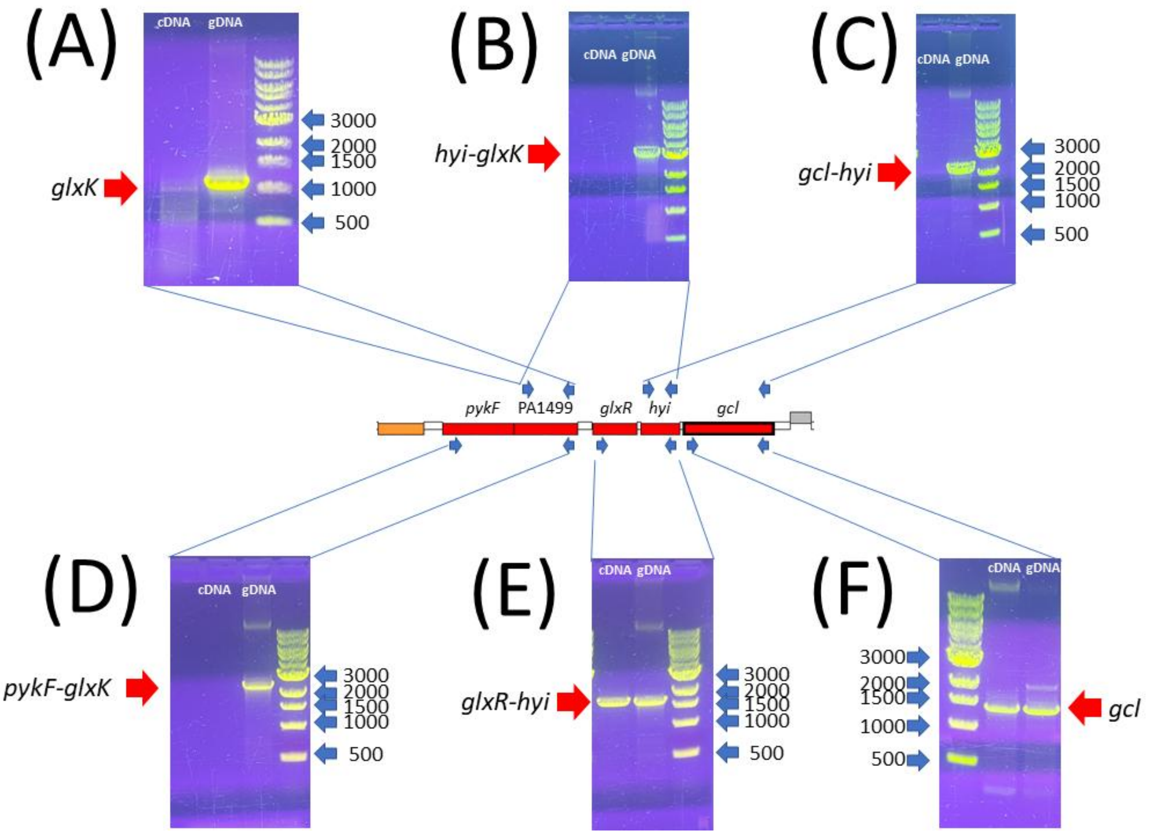

Figure S2. **Operonic structure of the *gcl* cluster of ORFs**. RNA was purified from allantoin-grown cultures of PAO1 and converted to cDNA. The indicated primers were then used to PCR-amplify the intervening stretches of DNA from either the cDNA template or a gDNA template, as indicated. The presence of a band of the indicated size (red arrows) in the lanes containing cDNA template indicates continuity in the mRNA sequence between the two primers (ie, presence of the associated ORFs on a common (shared) same transcriptional unit). The robust amplicons seen in lanes containing gDNA template indicates that the primers anneal well to the templates.

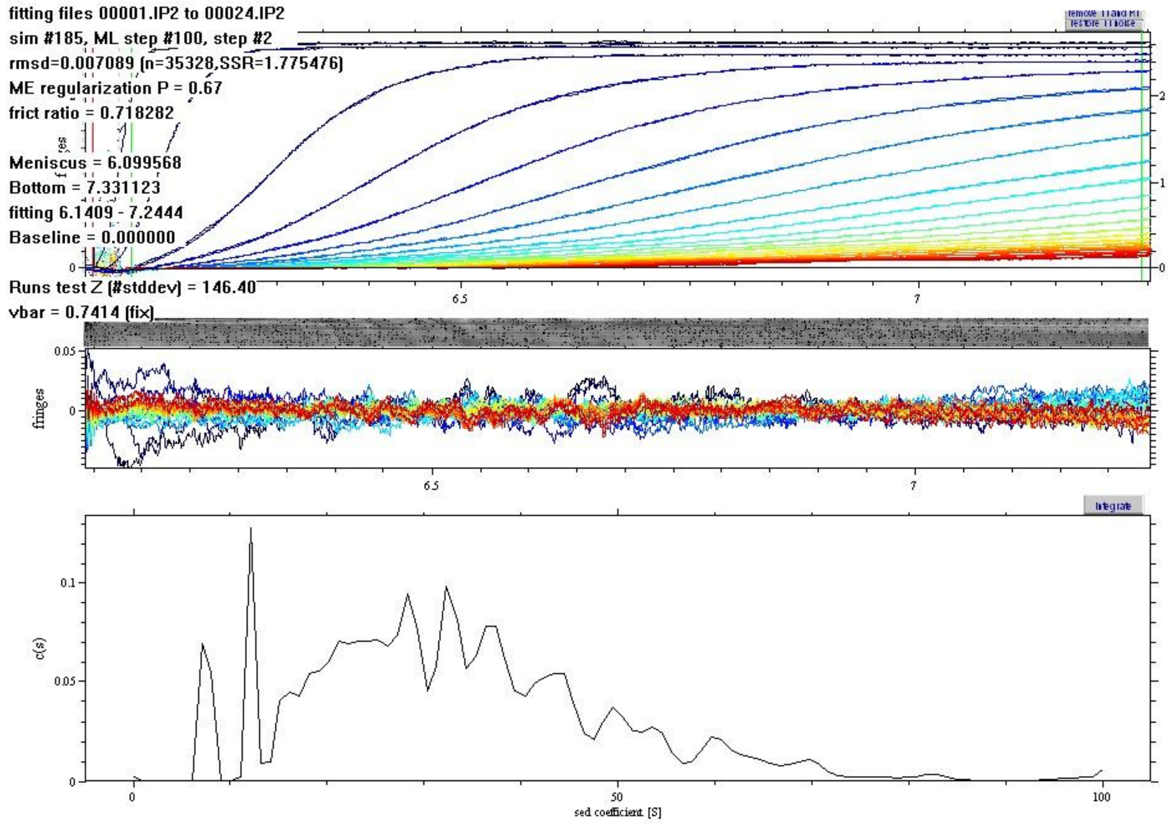

Figure S3. AUC-SV data for Gcl (64.6 kDa). Data does not indicate a specific multimeric state, instead showing a highly aggregated and polydisperse protein solution. The residuals are from the fit with the continuous c(s) distribution model. Species shown by component sedimentation coefficient distribution assuming a uniform frictional ratio of $F_{k,w}$ = 0.7. The final r.m.s.d. was 0.007. To prepare samples for AUC-SV, purified aliquots of Gcl were dialyzed against 20 mM Tris, 100 mM NaCl, 0.1 mM EDTA, 1 mM DTT (pH 7.5) at 4°C to remove any residual glycerol. For Gcl, 100 µM thiamine diphosphate, 10 µM FAD, and 10 mM coenzyme Q_0 were also present in the dialysis buffer to increase stability. After dialysis, the protein solution was clarified by centrifugation (20,000 × *g*, 10 min, 4°C). AUC-SV was performed with an Optima XL-I (Beckman Coulter) centrifuge and an An60 Ti four-hole rotor. Samples (400 µL of purified protein (c. 1 mg/mL) or matched buffer) were loaded into standard double-sector Epon centrepieces equipped with sapphire windows. Interference data were collected at 260 s intervals (50,000 rpm, 20°C). SEDNTERP was used to calculate buffer density and viscosity and the partial specific volume of the protein. Multi-component sedimentation coefficient distributions were obtained by direct boundary modelling of the Lamm equation using SEDFIT v.14.1 from 24 scans.

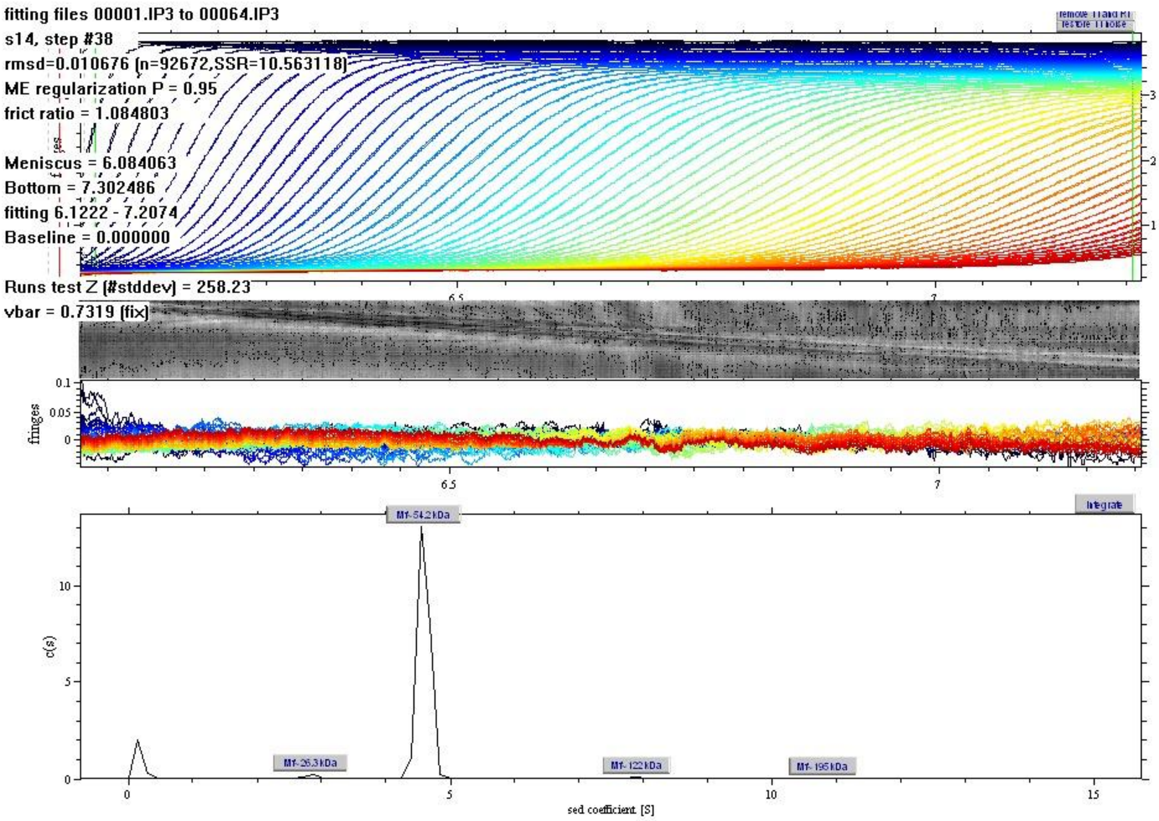

Figure S4. AUC-SV data for Hyi (31.3 kDa), indicating the protein is a dimer in solution (54.2 kDa). The residuals are from the fit with the continuous c(s) distribution model. Species shown by component sedimentation coefficient distribution assuming a uniform frictional ratio of $F_{k,w}$ = 1.08. The final r.m.s.d. was 0.011. The data were collected as outlined in Figure S3. Multi-component sedimentation coefficient distributions were obtained by direct boundary modelling of the Lamm equation using SEDFIT v.14.1 from 64 scans.

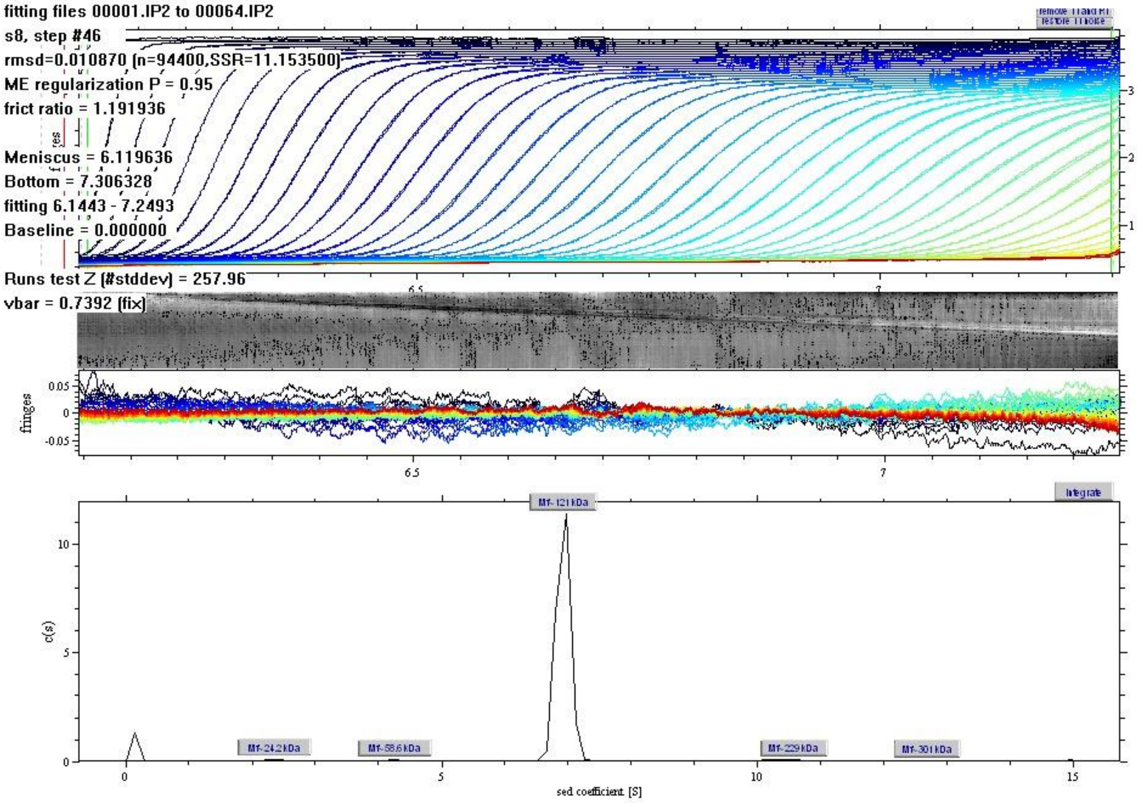

Figure S5. AUC-SV data for GlxR (33.0 kDa), indicating the protein is a tetramer in solution (121 kDa). The residuals are from the fit with the continuous c(s) distribution model. Species shown by component sedimentation coefficient distribution assuming a uniform frictional ratio of $F_{k,w}$ = 1.19. The final r.m.s.d. was 0.011. The data were collected as outlined in the legend to Figure S3. Multi-component sedimentation coefficient distributions were obtained by direct boundary modelling of the Lamm equation using SEDFIT v.14.1 from 64 scans.

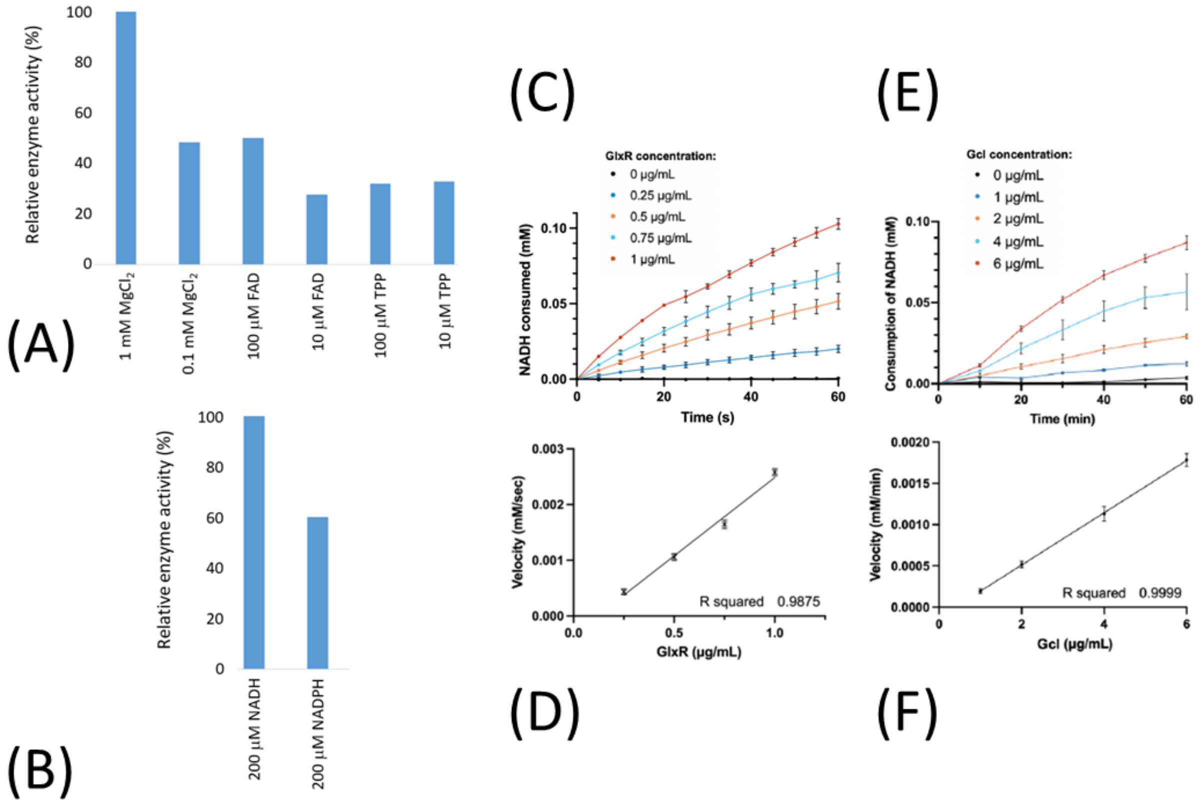

Figure S6. Effect of cofactors on Gcl activity. The figure shows the relative activity recorded in assay mixtures containing different amounts of the indicated cofactors. Standard assay mixtures contained 4 μg mL^{-1} Gcl, 20 μg mL^{-1} GlxR, 20 μg mL^{-1}, Hyi, 20 mM MOPS (pH 7.5), 1 mM glyoxylate, 100 μM $MgCl_2$, 200 μM NADH (or NADPH, as indicated), 100 μM FAD, and 10 μM TPP. However, in each of the reactions shown, [initial] concentrations of the highlighted cofactors (**A**) or nucleotide substrates (**B**) were adjusted to the indicated values, with all other reaction mixture components held at the starting concentrations shown in the standard assay mixture. The highest activity measurements made with either the different cofactors (**A**) or with the two different substrates (NADH or NADPH, (**B**) were designated as 100%, and activities in the presence of the other cofactors/substrates are shown relative to this. **TSA reductase activity is GlxR dependent**. The rate of NADH consumption on hydroxypyruvate as a substrate was negligible in the absence of GlxR (**C**) and increased in direct proportion to the amount of GlxR added (**D**). The rate of GlxR-catalyzed NADH oxidation was negligible in the absence of Gcl-derived TSA (**E**) and increased in direct proportion to the amount of Gcl present in the reaction mixture (**F**).
